# Understanding the malignant potential of gastric metaplasia of the oesophagus and its relevance to Barrett’s oesophagus surveillance: individual-level data analysis

**DOI:** 10.1136/gutjnl-2023-330721

**Published:** 2023-11-21

**Authors:** Emily L Black, Emma Ococks, Ginny Devonshire, Alvin Wei Tian Ng, Maria O’Donovan, Shalini Malhotra, Monika Tripathi, Ahmad Miremadi, Adam Freeman, Hannah Coles, Oesophageal Cancer Clinical and Molecular Stratification (OCCAMS) Consortium, Rebecca C Fitzgerald

**Affiliations:** 1 Early Cancer Institute, Department of Oncology, University of Cambridge, Cambridge, UK; 2 Cancer Research UK Cambridge Institute, University of Cambridge, Cambridge, UK; 3 Department of Histopathology, Cambridge University Hospitals NHS Foundation Trust, Cambridge, UK

**Keywords:** GASTRIC METAPLASIA, BARRETT'S METAPLASIA, BARRETT'S OESOPHAGUS, SURVEILLANCE, OESOPHAGEAL CANCER

## Abstract

**Objective:**

Whether gastric metaplasia (GM) of the oesophagus should be considered as Barrett’s oesophagus (BO) is controversial. Given concern intestinal metaplasia (IM) may be missed due to sampling, the UK guidelines include GM as a type of BO. Here, we investigated whether the risk of misdiagnosis and the malignant potential of GM warrant its place in the UK surveillance.

**Design:**

We performed a thorough pathology and endoscopy review to follow clinical outcomes in a novel UK cohort of 244 patients, covering 1854 person years of follow-up. We complemented this with a comparative genomic analysis of 160 GM and IM specimens, focused on early molecular hallmarks of BO and oesophageal adenocarcinoma (OAC).

**Results:**

We found that 58 of 77 short-segment (*<*3 cm) GM (SS-GM) cases (75%) continued to be observed as GM-only across a median of 4.4 years of follow-up. We observed that disease progression in GM-only cases and GM+IM cases (cases with reported GM on some occasions, IM on others) was significantly lower than in the IM-only cases (Kaplan-Meier, p=0.03). Genomic analysis revealed that the mutation burden in GM is significantly lower than in IM (p<0.01). Moreover, GM does not bear the mutational hallmarks of OAC, with an absence of associated signatures and driver gene mutations. Finally, we established that GM found adjacent to OAC is evolutionarily distant from cancer.

**Conclusion:**

SS-GM is a distinct entity from SS-IM and the malignant potential of GM is lower than IM. It is questionable whether SS-GM warrants inclusion in BO surveillance.

**WHAT IS ALREADY KNOWN ON THIS TOPIC**

- The UK guidelines are distinct from most countries, by not requiring goblet cells (intestinal metaplasia (IM)) for a diagnosis of Barrett’s oesophagus.
- IM can be misdiagnosed as gastric metaplasia (GM) if sampling is insufficient.

**WHAT THIS STUDY ADDS**

- Short-segment GM can be molecularly distinguished from short-segment IM.
- The risk of progression is substantially lower in GM than in IM.
- GM does not bear the genomic hallmarks of oesophageal adenocarcinoma.
- GM is not evolutionarily close to oesophageal adenocarcinoma, even when found adjacent to the cancer.

**HOW THIS STUDY MIGHT AFFECT RESEARCH, PRACTICE OR POLICY**

- The low risk of progression in GM suggests that UK Barrett’s oesophagus surveillance guidelines could be updated to require the presence of goblet cells in short segments.
- This would reduce oversurveillance, resulting in better quality of life for these patients and improved focusing of resources on higher risk patients.
- It would also bring the UK in line with the international community. This would reduce confusion, and facilitate comparisons, for research and clinical practice.

## Introduction

Barrett’s oesophagus (BO) is a precursor lesion for oesophageal adenocarcinoma (OAC) and provides an opportunity to improve outcomes by detecting dysplasia and cancer early. BO is characterised by the replacement of squamous oesophageal epithelium with columnar-lined epithelium with a crypt architecture. Metaplastic glands in BO can be gastric or intestinal in nature, and a non-dysplastic BO (NDBO) segment will typically be a mosaic of different gland types. A segment with only gastric-type glands (no goblet cells) is called gastric metaplasia (GM); identification of any intestinal-type glands leads to a designation of intestinal metaplasia (IM) (online supplemental figure 1). NDBO can progress to low-grade and high-grade dysplasia (LGD, HGD) and then potentially to OAC.

10.1136/gutjnl-2023-330721.supp1 Supplementary data

Most patients with BO will never develop OAC though, with annual progression rates to OAC of 0.1%–0.5% for NDBO.^1–3^ The UK surveillance guidelines stand apart in their definition of BO. In the UK, GM of at least 1 cm is sufficient for a BO diagnosis^4^; elsewhere, presence of IM is required.^5–8^ This distinction may be substantial: a study suggested that removing the US requirement for goblet cells would lead to a 147% increase in BO diagnoses, with little impact on OAC diagnoses.^9^

**Figure 1:**
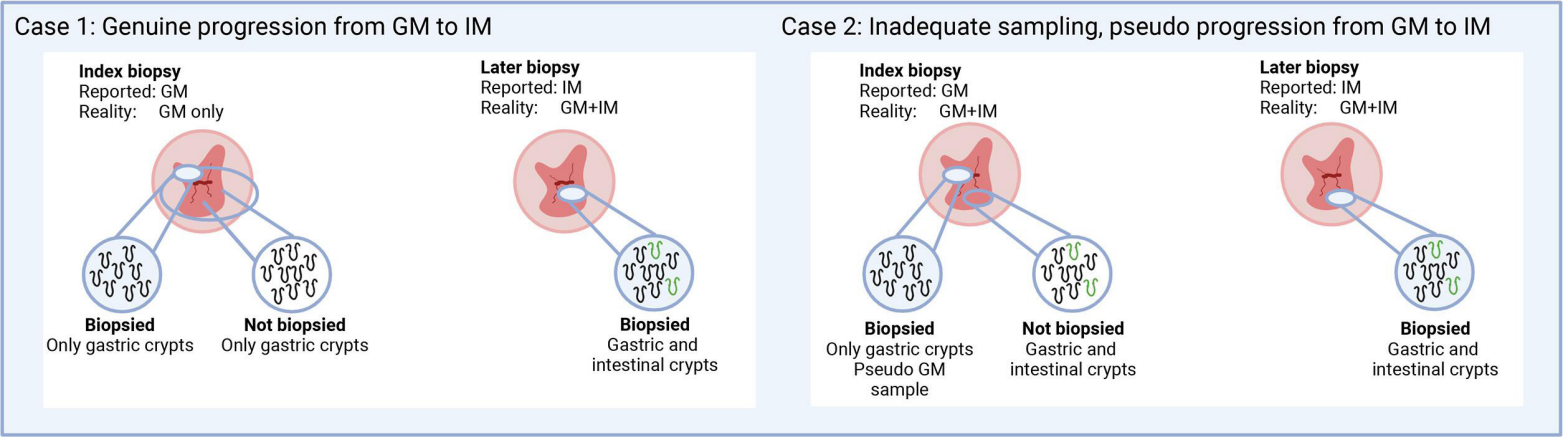
Illustration of the challenge of misdiagnosis due to undersampling of GM+IM segments. BO, Barrett’s oesophagus; GM, gastric metaplasia; IM, intestinal metaplasia.

However, there remains conflicting clinical evidence about the malignant potential of GM. While there is evidence supporting GM having very low risk of progression, and lower risk than IM,^9–11^ several studies reached an opposing conclusion. Specifically, that most GM cases progressed to IM^12 13^ and then on to OAC at a similar rate as index-IM cases.^14^ Much of the discrepancy is likely due to sampling. In patients with gastric and intestinal glands (GM+IM), goblet cells are not uniformly distributed,^15^ so areas of the segment can look like GM and be misdiagnosed (figure 1).

Frequent co-occurrence of GM and OAC has been used to argue the malignant potential of GM and to contest the view that OAC typically arises from IM.^16^ However, overgrowth of IM and sampling error could also explain the absence of visible IM.^17 18^ Recent computational analysis provided new evidence that most OACs do arise via IM, despite its frequent absence.^19^ Furthermore, it has been shown that IM develops from undifferentiated gastric cardia cells, and that BO is a unifying pathway to OAC.^20^ Transcriptional differences suggest that true GM is more akin to normal gastric cells than IM.^20–23^

The molecular hallmarks of OAC are single base substitution (SBS) signatures 17a and 17b, *TP53* mutations, genomic instability, a plethora of lower frequency mutations and larger scale structural rearrangements.^24–26^ Evidence of genomic differences between GM and IM is poorly delineated and tends to be based on a small number of samples and a subset of the genome.^27 28^ Reported phylogenies vary from cases where GM shares little evolutionary history with OAC,^29^ to a case where OAC had developed directly from gastric BO glands.^30^

**Figure 2:**
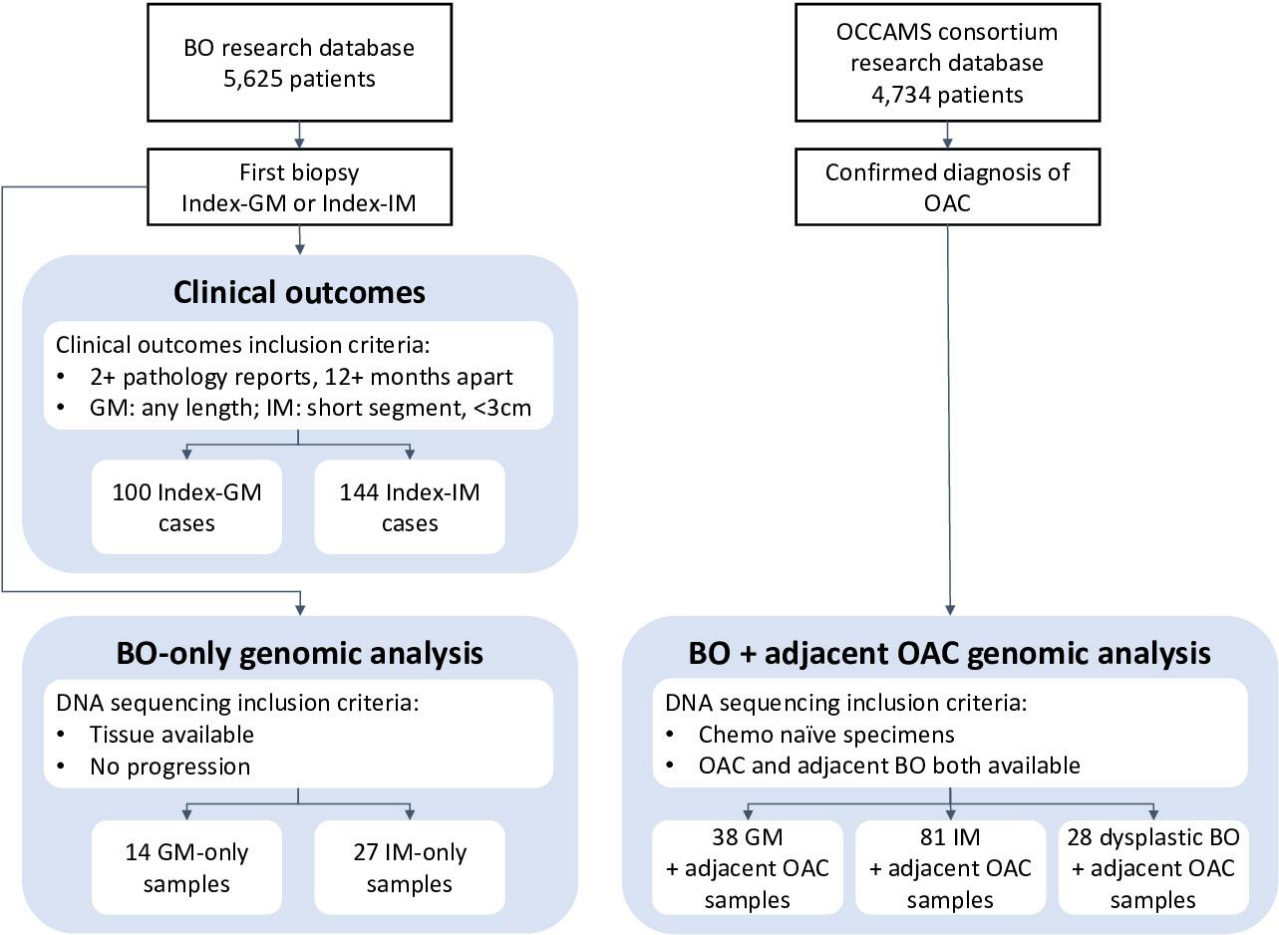
Study design. Flow chart showing creation of clinical outcomes cohort and sequencing cohorts. BO, Barrett’s oesophagus; GM, gastric metaplasia; IM, intestinal metaplasia; OAC, oesophageal adenocarcinoma.

This study examines the relative malignant potential of GM compared with IM, to assess whether the distinction between the two is clinically relevant. We performed a thorough review of endoscopy and pathology reports across a novel cohort of 244 patients (figure 2), covering 1854 person years of follow-up. We separately assessed outcomes in GM-only, IM-only and GM+IM cases. We complemented clinical outcome analysis with genomic analysis assessing molecular hallmarks of OAC in GM and IM, in both BO only and BO adjacent to OAC contexts.

## Methods

### Clinical outcome cohort

A cohort was selected from a database of patients under BO surveillance at Addenbrooke’s Hospital (Cambridge University Hospitals NHS Foundation Trust) who consented prospectively to research participation. Strict selection criteria were applied to pathology, endoscopy and medical histories (online supplemental table S1). All oesophagogastroduodenoscopies (OGDs) comprised of multiple biopsies, each with known pathology. The centre follows a systematic biopsy protocol, in-line with the Seattle protocol. Each OGD had a single classification of IM (if any biopsy had IM) or GM (if no biopsy had IM). Patients needed to have an index diagnosis of GM or IM. The index segment length needed to be at least 1 cm, with no upper limit for index-GM cases, but less than 3 cm for index-IM cases. From a total of 5625 patients in the database, 244 were selected: 77 index-GM, short-segment cases; 23 index-GM, long segment; 144 index-IM, short segment (figure 2).

### Surveillance analysis

Cases with no follow-up after 1 December 2016 were presumed to have been discharged. This represented 6 years at the time of analysis. IM biopsies were classified into one of three groups: focal IM, widespread IM or unspecified, based on pathologist judgement. The terms ‘focal’ or ‘minimal’ were considered for focal IM, and ‘widespread’ or ‘extensive’ for widespread IM. Cases were classified as GM-only or IM-only, if OGD diagnoses were consistent; GM+IM, if there were both GM and IM diagnoses in the case history. If an otherwise IM-only case-reported GM followed by IM less than a year later, this was considered IM-only.

### BO-only genomic cohort

Tissue samples were available for patients in the Cell Determinants Biomarker study (REC 01/149), an observational study to identify biomarkers of the development and progression of BO. GM-only patients had to have only ever had GM biopsies and were chosen to include a range of ages and both male and female; 14 patients were selected.^21^ Twenty-seven ‘non-dysplastic, non-progressor’ cases formed an IM-only comparative set^31^ (figure 2).

### Adjacent BO and OAC genomic cohort

A cohort was selected from patients with tissue samples available from OAC resections or biopsies. The patients were sourced from the Oesophageal Cancer Classification and Molecular Stratification (OCCAMS) consortium in the UK. Individual informed consent was provided by all subjects (REC 07/H0305/52 and 10/H0305/1). Whole genome sequencing (WGS) was performed on 53 IM, 4 GM and 28 dysplasia samples and on OAC adjacent to each. Whole exome sequencing (WES) was performed on 34 GM and 28 IM samples, from 54 patients (8 patients had GM and IM), along with adjacent OAC, as well as 42 OAC samples with no adjacent BO (figure 2). Patients had to be chemo-naïve at the time of sampling. Patient characteristics are included in the online supplemental table S2.

### DNA extraction, library preparation and sequencing

For WGS, genomic DNA from samples and germline controls was extracted and processed as previously reported.^21 31^ For WES, samples were prepared from formalin-fixed paraffin-embedded (FFPE) slides. Pathologists identified areas of distinct tissue type with sufficient cellularity for sequencing (online supplemental figure 5), which were macrodissected and processed as described in the online supplemental methods.

Library preparation and enrichment for the WES samples were performed using Illumina DNA Prep with Enrichment, with 6-plex pooling. Sequencing was performed on an Illumina NovaSeq 6000 or HiSeq 4000, at the Cancer Research UK Cambridge Institute. Mean sequencing depth was 150×, with germline samples sequenced to at least 33× and BO or OAC samples to at least 53×.

### Variant calling and copy number alterations

For the WGS samples, variants, copy number alterations (CNAs) and mutational signatures were called as previously described.^21 31^ For the WES samples, analysis focused on single-nucleotide variants (SNVs) and small insertion and deletions (indels). FASTQ files were aligned to GRCh37 using BWA-MEM, with duplicates marked by Picard V.2.9.5. Variant calling was performed using GATK Mutect2 V.4.1.7.0,^32^ using multisample and FFPE settings. Mutation filtering and copy number analysis are detailed in the online supplemental methods.

### Driver gene analysis

The set of OAC driver genes used was the 76 genes identified by Frankell *et al*.^25^ We classed a subset of these as ‘early IM/OAC genes’: *TP53, CDKN2A, ARID1A, SMARCA4, MUC6*. These genes are frequently mutated in IM,^31 33 34^ and under selection in IM.^35^ Only non-silent mutations were included in driver gene analysis. Where multiple samples of the same tissue type were extracted for the same patient, a single sample was chosen at random to be included in analyses.

### Phylogenetic analysis

Clustering of SNVs was performed in PyClone V.0.13.1,^36^ integrating CNAs. Identified clusters with at least 10 mutations and median variant allele frequency (VAF) of at least 0.05 were used in ClonEvol^37^ to create phylogenetic trees, with indels assigned to clusters post hoc. Shared mutations between the BO and OAC, with cancer cell fraction of at least 0.3 in OAC, were set as the founding cluster, even if fewer than 10 mutations.

### Statistical analysis

Statistical analysis was performed in R V.4.0.3. The ‘survival’ and ‘survminer’ packages were used for Kaplan-Meier analysis and survival plots; with p values from log-rank tests. For mutational burden and somatic chromosomal alteration (SCA) load, means were compared using unpaired Wilcoxon’s tests. A Pearson’s χ^2^ test tested association between focal and widespread IM and outcomes. For all statistical analyses, a p value <0.05 was considered significant. STROBE (Strengthening the Reporting of Observational Studies in Epidemiology) cohort reporting guidelines were used.^38^

### Patient and public involvement

Patient group Heartburn Cancer UK review and co-design all our patient facing materials for the Cell Determinants Biomarker and OCCAMS studies. A lay friendly version of this paper will be shared with patients and the public.

## Results

### Short-segment GM is a distinct state, not only mis-sampling of IM

To understand the prevalence of true GM compared with mis-sampling of GM+IM, we reviewed the full pathological history for 100 index-GM cases. Seventy-seven were short segments (SS, *<*3 cm) on index endoscopy and 23 were long segments (LS, *≥*3 cm). Of the 77 index-GM-SS cases, over a median follow-up of 6.1 years, the majority (58 cases, 75%) were found to have a GM-only outcome (table 1). These GM-only outcomes confirm that persistent GM can be a distinct state in short segments.

**Table 1:** Outcomes across all biopsy results for each of the three index biopsy subgroups

All biopsy results	Index GM-SS	Index GM-LS	Index IM-SS	Total cases	Mean OGDs
GM only	58 (75%)	9 (39%)		**67**	3.28
*of which progressed to LGD*	0	0		0	
*of which progressed to HGD or IMC*	0	0		0	
GM+IM	19 (25%)	14 (61%)	44 (31%)	**77**	4.84
*of which progressed to LGD*	1	0	1	2	
*of which progressed to HGD or IMC*	0	0	0	0	
IM only			100 (69%)	**100**	4.20
*of which progressed to LGD*			6	6	
*of which progressed to HGD or IMC*			5	5	
Total	77	23	144	**244**	4.15
Median follow-up years	6.1	9.7	7.8	7.2	
Total follow-up years	473	220	1161	1854	

GM, gastric metaplasia; HGD, high-grade dysplasia; IM, intestinal metaplasia; LGD, low-grade dysplasia; OGDs, ophagogastroduodenoscopies.

In contrast to short segments, in the majority of index-GM-LS cases (14/23 cases, 61%), IM was detected in at least one later biopsy. Later detection of IM could be due to sampling at index or progression, and these two scenarios cannot be distinguished here. Four of the 14 GM+IM cases were first diagnosed before the Seattle protocol was recommended. A GM+IM outcome was also noted in 44 of the 144 (30%) index-IM-SS cases (table 1).

In some pathology reports, IM was noted as focal or widespread. This was examined to understand if focal IM contributed to GM+IM outcomes. A Pearson’s χ^2^ test showed that a GM+IM versus IM-only outcome was significantly dependent on focal or widespread IM (online supplemental table S3).

**Figure 3:**
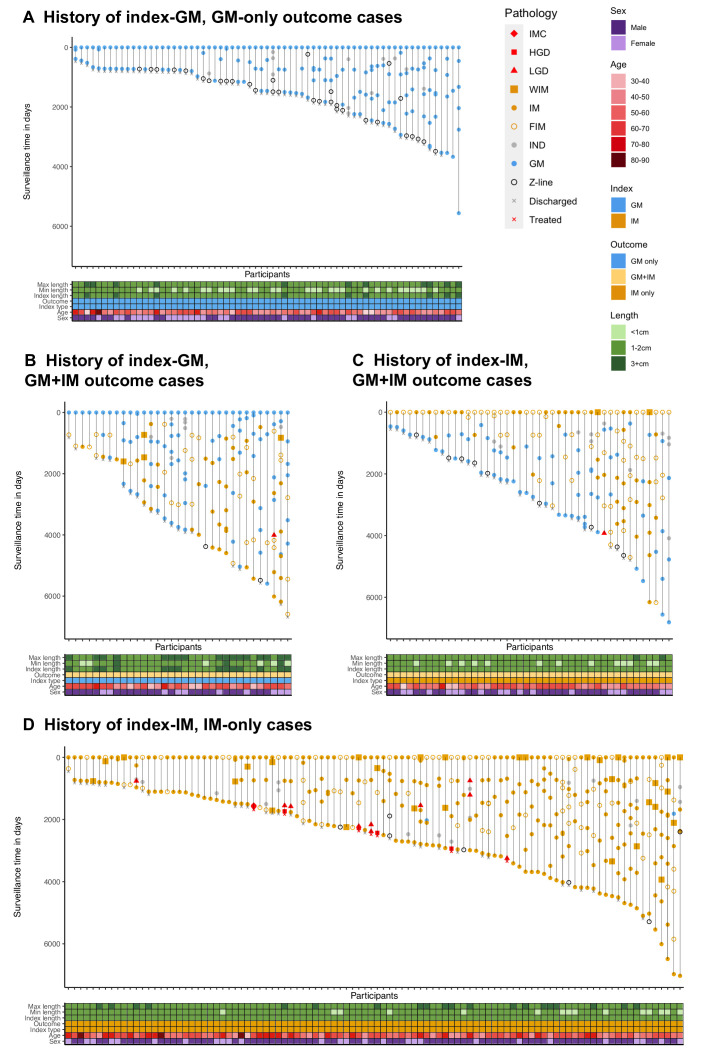
GM is a distinct state, not mis-sampling of IM. Progression to dysplasia and cancer is lower in GM than IM. (A) Surveillance history of 67 index-GM, GM-only outcome cases. (B) Surveillance history of 33 index-GM, GM+IM outcome cases. (C) Surveillance history of 44 index-IM, GM+IM outcome cases. (D) Surveillance history of 100 index-IM, IM-only outcome cases. For (A−D), each vertical trajectory is an individual case history, with the marker shapes denoting the histopathological assessment for each OGD. Each case has its surveillance time indexed to baseline, with the length of the vertical line representing total surveillance time. BO, Barrett’s oesophagus; FIM, focal intestinal metaplasia; GM, gastric metaplasia; HGD, high-grade dysplasia; IM, intestinal metaplasia; IMC, intramucosal carcinoma; IND, indefinite for dysplasia; LGD, low-grade dysplasia; WIM, widespread intestinal metaplasia.

The sequence of diagnoses within the 33 index-GM, GM+IM outcome cases, was reviewed for patterns that could inform clinical practice, but no consistent patterns were observed (figure 3B, online supplemental table S4). Eighteen cases (55%) required three or more OGDs before observing IM. If a GM+IM outcome is due to sampling, a single repeat OGD is not always sufficient to overcome this challenge.

The surveillance history of the index-IM, GM+IM cases was limited, as 17 of the 44 patients (39%) had no further OGDs after the first GM or irregular Z-line observation (figure 3C). The surveillance histories of GM-only and IM-only cases are included for reference (figure 3A and D).

### Progression to dysplasia and cancer is lower in GM and GM+IM than IM

The progression rates to dysplasia and OAC were compared between GM, GM+IM and IM to understand the malignant risk. In total, this covered 1854 person-years of follow-up, with a median of 4.9 years in GM-only cases, 10.1 years in GM+IM cases and 7.5 years in IM-only cases. There was no progression to LGD, HGD or cancer in GM-only cases. Progression to LGD was identified in one index-GM, GM+IM case (1%), but no progression to HGD or cancer. This case that progressed was not treated after the LGD diagnosis, showed no abnormal p53 expression, and LGD was not seen again on later biopsies (figure 3B). Re-review by an expert upper gastrointestinal pathologist upheld the LGD diagnosis. Progression was higher within index-IM cases. Seven index-IM cases (5%, one GM+IM, six IM-only) progressed to LGD but not further. Of these seven cases, three were treated (as now recommended by guidelines) and four were not. Recent biopsies for the untreated cases did not show LGD, consistent with the difficulty in diagnosing this grade of dysplasia. A further five index-IM cases (3%, all IM-only) progressed to HGD or IMC (table 1).

**Figure 4:**
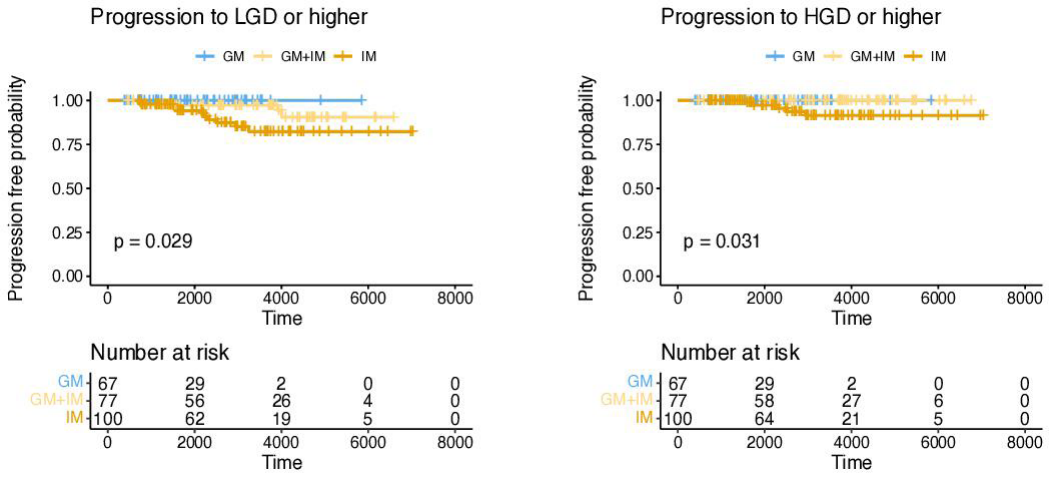
Progression to dysplasia and cancer is lower in GM than IM. Kaplan-Meier curves for progression to LGD or higher and to HGD or higher, separated by classification. GM, gastric metaplasia; HGD, high-grade dysplasia; IM, intestinal metaplasia; LGD, low-grade dysplasia.

Kaplan-Meier analysis compared progression to LGD and HGD in GM-only, GM+IM and IM-only cases (figure 4). For both LGD and HGD, there was a significance in the difference between progression rate by metaplasia type (p=0.029 LGD, p=0.031 HGD), with progression lower in GM and GM+IM than in IM.

Finally, to understand consistency in length assessment, the endoscopy history was reviewed for each case. Strikingly, nearly all index GM-LS cases had a shorter length on a later endoscopy. The converse pattern was not observed: segments were rarely reported as a longer length in later OGDs (online supplemental figure 2). This suggests a trend over time to shorter assessments, rather than natural variation in length assessment.

### GM bears few genomic hallmarks of OAC

**Figure 5:**
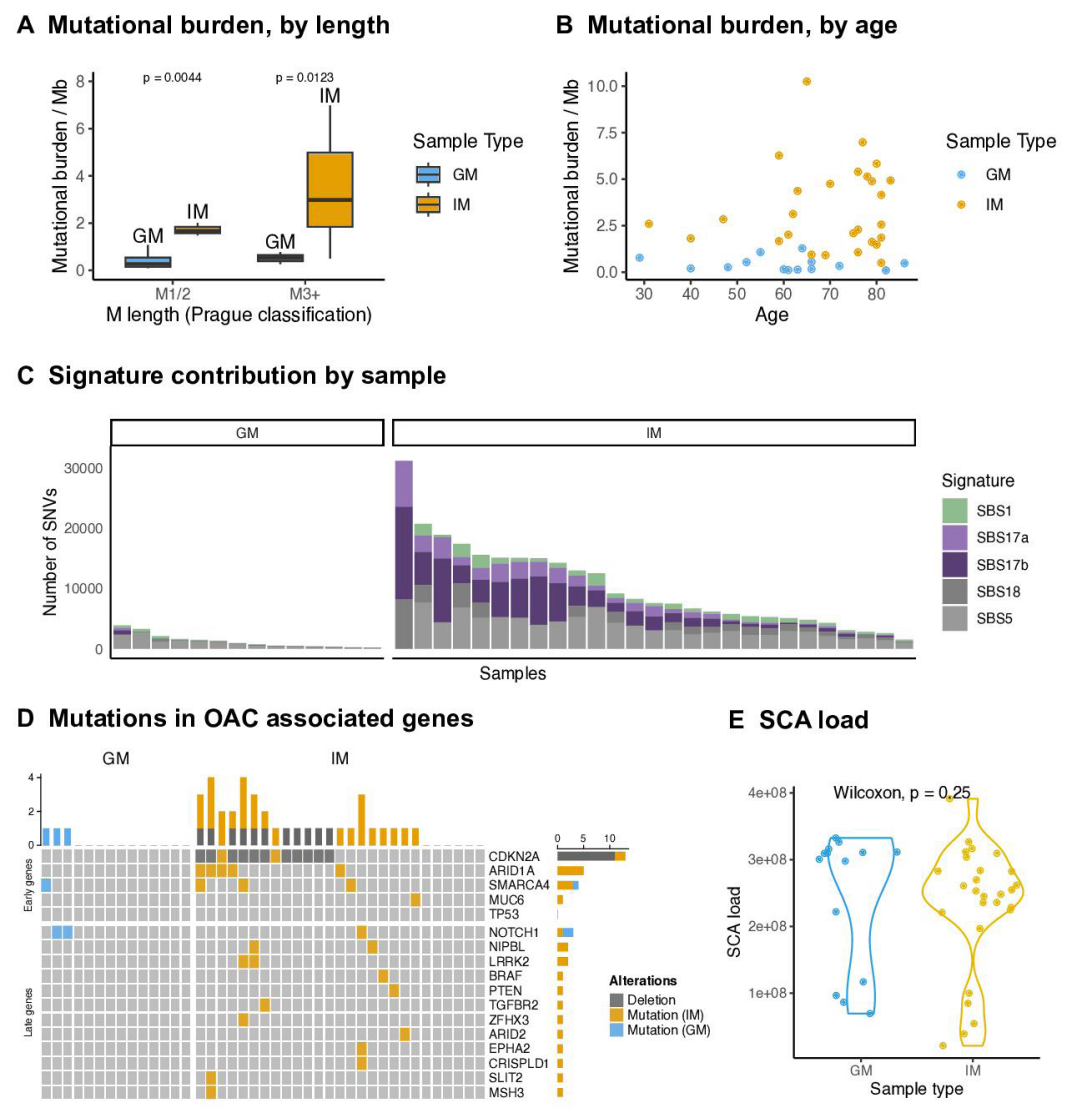
GM does not bear the genomic hallmarks of OAC. (A) Mutational burden by sample type and BO segment length. (B) Mutational burden by age. (C) SBS signature contribution by sample. (D) Mutations in OAC associated genes, split by genes typically mutated early and late in the progression of OAC. Only genes with mutations in this cohort, along with TP53, are shown. (E) SCA load by sample, taken as the length of genome altered by a copy gain, loss or copy neutral loss of heterozygosity. BO, Barrett’s oesophagus; GM, gastric metaplasia; IM, intestinal metaplasia; OAC, oesophageal adenocarcinoma; SCA, somatic chromosomal alterations; SNV, single nucleotide variant.

It is well known that IM bears genomic hallmarks of OAC, such as driver gene mutations and signatures SBS17a/SBS17b. Less has been reported on the genomics of GM, particularly in a GM-only context. In the WGS of 14 GM-only samples and 27 IM-only samples, a significantly lower mutational burden in GM compared with IM was evident, even when the samples were partitioned into short and long segments (figure 5A). The difference in burden was also not explained by the ages in the two groups (figure 5B). SBS17a/b were detected in nearly all IM samples, but only in one GM sample (figure 5C). SBS1, a signature associated with ageing, was present in all GM samples, and all but one IM sample.

All samples were assessed for non-silent mutations and CNAs in genes associated with IM and OAC. Only one GM sample (7%) had an alteration in an early IM/OAC gene, *SMARCA4*. By contrast, 16 IM samples (59%) had an alteration in at least one early IM/OAC gene (figure 5D). The SCA load—the length of the genome with a copy number gain, loss or loss of heterozygosity—was also assessed. There was no difference in SCA load between GM and IM non-progressors (figure 5E), and both were an order of magnitude lower than in OAC samples (online supplemental figure 3). No driver amplifications or deletions were seen in the GM samples.

### Co-occurring GM and OAC are genomically distant from one another

**Figure 6:**
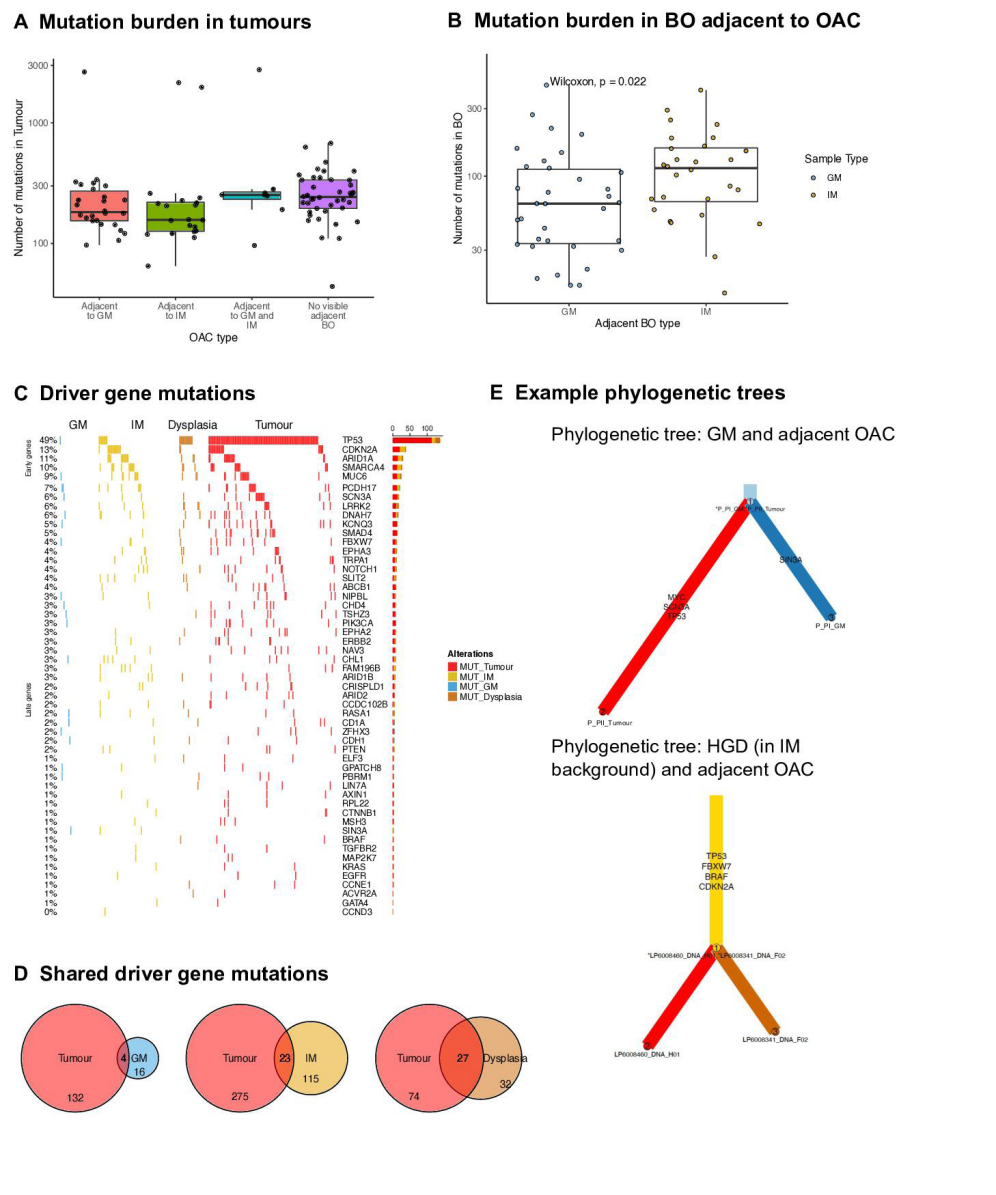
Co-occurring GM and OAC are evolutionarily distant from one another. (A) Mutation burden in OAC tumours, by type of adjacent BO. (B) Mutation burden in BO adjacent to OAC. (C) Mutations in driver genes by sample, across BO samples and adjacent OAC. Only genes with a mutation in a BO sample are shown. (D) Venn diagrams showing the degree of overlap in mutations between different types of BO and the adjacent OAC. (E) Example phylogenetic trees for one case with GM and adjacent OAC, and one case with HGD in an intestinal background, and adjacent OAC. Branch length represents number of mutations. BO, Barrett’s oesophagus; GM, gastric metaplasia; IM, intestinal metaplasia; OAC, oesophageal adenocarcinoma.

To better understand the evolutionary relationship between different types of BO and OAC, the mutations found in adjacent BO and tumours were assessed using WES and WGS. The mutational burden in tumours was independent of the presence or type of BO (figure 6A). In adjacent BO, the mutational burden varied by type of BO, with the burden significantly lower in adjacent GM than adjacent IM (p=0.022) (figure 6B).

We found that one adjacent GM sample (3%) had a mutation in *TP53* and one (3%) in *MUC6*, but no other GM samples had any mutations in early IM/OAC genes (figure 6C). By contrast, 41 (51%) IM samples and 19 (68%) dysplastic samples had mutations in early IM/OAC genes. Across the full set of OAC driver genes, on average, GM had mutations in 0.5 drivers, compared with 1.6 in IM and 2.0 in dysplasia. The one GM sample with a *TP53* mutation did not have a second hit on *TP53* and had a low VAF of 0.11. Of the 9 IM samples with *TP53* mutations, 4 (44%) had a second hit in the form of LOH, as did 10 of the 14 (71%) *TP53*-mutated dysplastic samples.

There were very few shared driver gene mutations between the 38 GM samples and their adjacent OAC: 4 shared, 16 unique to GM, 132 unique to OAC (figure 6D). In the WGS samples, there were no driver amplifications or deletions called in GM. It is notoriously difficult to robustly call CNAs in WES, but using a joint segmentation approach, one shared amplification was called between GM and OAC (online supplemental figure 4), although in a GM sample with no *TP53* mutation. Mutational overlap was also low between IM and OAC, but much higher between dysplasia and OAC (27 shared, 32 unique to dysplasia, 74 unique to OAC, figure 6D). This suggests that GM is distantly related to the OAC, and most mutations in driver genes in GM are not drivers of oncogenesis in this context. Phylogenetic trees for the matched samples, with branch length representing number of mutations, also demonstrate the relative distance of GM from OAC compared with dysplasia and OAC (figure 6E). The example tree with GM and OAC had a very short trunk of shared mutations and no shared OAC driver gene mutations. By contrast, the HGD and OAC tree had a long trunk, including four shared mutations in OAC driver genes.

## Discussion

We present clinical and genomic evidence for the lower malignant potential of GM. We followed clinical outcomes in a novel cohort of 244 patients, uniquely representing recent clinical practice in a country where GM is still surveilled. We also analysed sequencing of 41 GM and IM samples from non-progressors and 119 GM and IM samples from patients with OAC.

The clinical data demonstrate that the progression in GM and GM+IM is extremely low and lower than in IM (0% incidence per year of HGD or cancer in index-GM, 0.43% in index-IM). This is consistent with a large-scale population study,^10^ which observed a lower rate of progression in 3179 GM cases compared with 3917 IM cases (0.07% vs 0.38% incidence of HGD or cancer per year) but lacked length data and a systematic biopsy protocol. The results are also consistent with the US study by Westerhoff *et al*,^9^ which saw no progression to OAC across 379 patients with GM but did not have a focus on patients with GM+IM. While it cannot be said that GM will never progress to OAC, we expect that the progression rate is more akin to that of the general population than that of patients with IM.

The results may appear inconsistent with the study by Evans *et al*, which categorised BO segments on a microscopic level, based on diversity of gland phenotypes.^39^ They reported that segments with higher gland diversity had higher progression risk, which might be taken to suggest higher progression in GM+IM cases. However, there is no link between our longitudinal outcome categories and microscopic diversity of glands; IM-only segments can have higher diversity of glands than GM+IM segments, especially given IM is so often focal within GM+IM cases.

It is well known that IM can be missed in GM+IM samples, particularly if the IM is focal. However, most short GM segments continued to receive a GM diagnosis, including cases with over five OGDs. This gives confidence that these are true GM segments throughout the surveillance period, not undersampled GM+IM. This is consistent with the findings of Chandrasoma *et al*,^11^ who found that under a robust sampling regime, it was possible to identify true GM cases, and Westerhoff *et al*, who found that 88% of purported patients with GM continued to have no goblet cells.^9^ While other studies have shown much higher rates of later IM in index-GM cases,^12–14^ much can be explained by inadequate sampling, prior to the introduction of the Seattle protocol.

By contrast, the majority of the long GM segments were found to contain IM in a later biopsy, consistent with a study where 7 out of 11 LS-GM were found to have IM later.^12^ Goblet cell density being higher proximally^15^ also ties with a higher likelihood of IM in a longer segment. These results suggest that more considered management of LS-GM cases is required than that of short-segment GM (SS-GM), as is already the case today.

The WGS of non-progressors showed that mutational burden in GM was significantly lower than that of IM (SS: p<0.01, LS: p=0.0123). In GM, there were very few mutations in genes associated with OAC, and signatures SBS17a/b were not prevalent. Altogether, GM does not bear the mutational hallmarks of OAC, but IM does. Similar results were seen in sequencing of GM and IM adjacent to OAC. These results are consistent with the targeted sequencing of Bandla *et al* 27 but strengthen the findings due to examining the whole genome or exome and almost four times as many OAC-associated genes.

Liu *et al* found similar levels of DNA-content abnormalities in GM and IM using image cytometry,^28^ and we too found similar SCA loads in GM and IM. Non-progressor IM, with no *TP53* mutation, rarely has substantial CNAs. Therefore, we do not consider this indicative of malignant potential in either metaplasia type.

Of the few driver gene mutations seen in adjacent-GM, there was little overlap with mutations in the tumour. Although Lavery *et al* showed that OAC could arise from gastric glands,^30^ this was a single case, with IM. It has not been shown whether this occurs widely or without IM present. Our results suggest that adjacent GM is not routinely evolutionarily closer than IM is to OAC. This is consistent with phylogenies presented by Bao *et al*.^29^ Since an argument for the malignant risk of GM is its co-occurrence with OAC, it is important to be clear that even when GM or IM is spatially close to OAC, it does not follow that the GM or IM is evolutionarily close to OAC. Instead, it is likely that the dysplastic clone the OAC directly evolved from has been overgrown or was not sampled.

A few limitations should be considered with the sequencing. First, while the macrodissection was carried out with great care, there remains the possibility that some GM areas could have been sampled from normal cardia. Second, mutations in GM occur at low-variant allele frequencies, so at standard sequencing depths, it is possible that there were undetected mutations in relevant genes. A real strength in the genomic analysis here is that BO-only and BO adjacent to OAC have both been studied, and the results are similar in the two contexts.

Many GM studies, including ours, suffer from small sample sizes, primarily due to few GM patients being enrolled in studies. A strength of our study is the number of GM+IM cases, as low progression in these should reassure clinicians concerned by misdiagnosing GM in a GM+IM case. As with the macrodissections, we cannot rule out some of the GM diagnoses in the clinical outcome cohort coming from mis-sampled cardia. Finally, we recognise the limitation of the outcome cohort coming from a single centre. However, since this is an expert centre that follows a systematic biopsy protocol and sees many tertiary referrals, we are confident in the robustness of our findings.

While not a focus of the study, the data suggest that focal IM has low progression risk. It has previously been shown that focal IM at the gastro-oesophageal junction, after radiofrequency ablation, does not progress to dysplasia.^40^ Confirmation of low progression in focal IM could further improve surveillance targeting. Systematic capture by pathologists, which is missing from our study, perhaps aided by digital pathology and AI, could inform this. Alternatively, the cytosponge device could assist, as quantification of TFF3 expression from a cytosponge sample can identify focal IM pathologies.^41^

**Figure 7:**
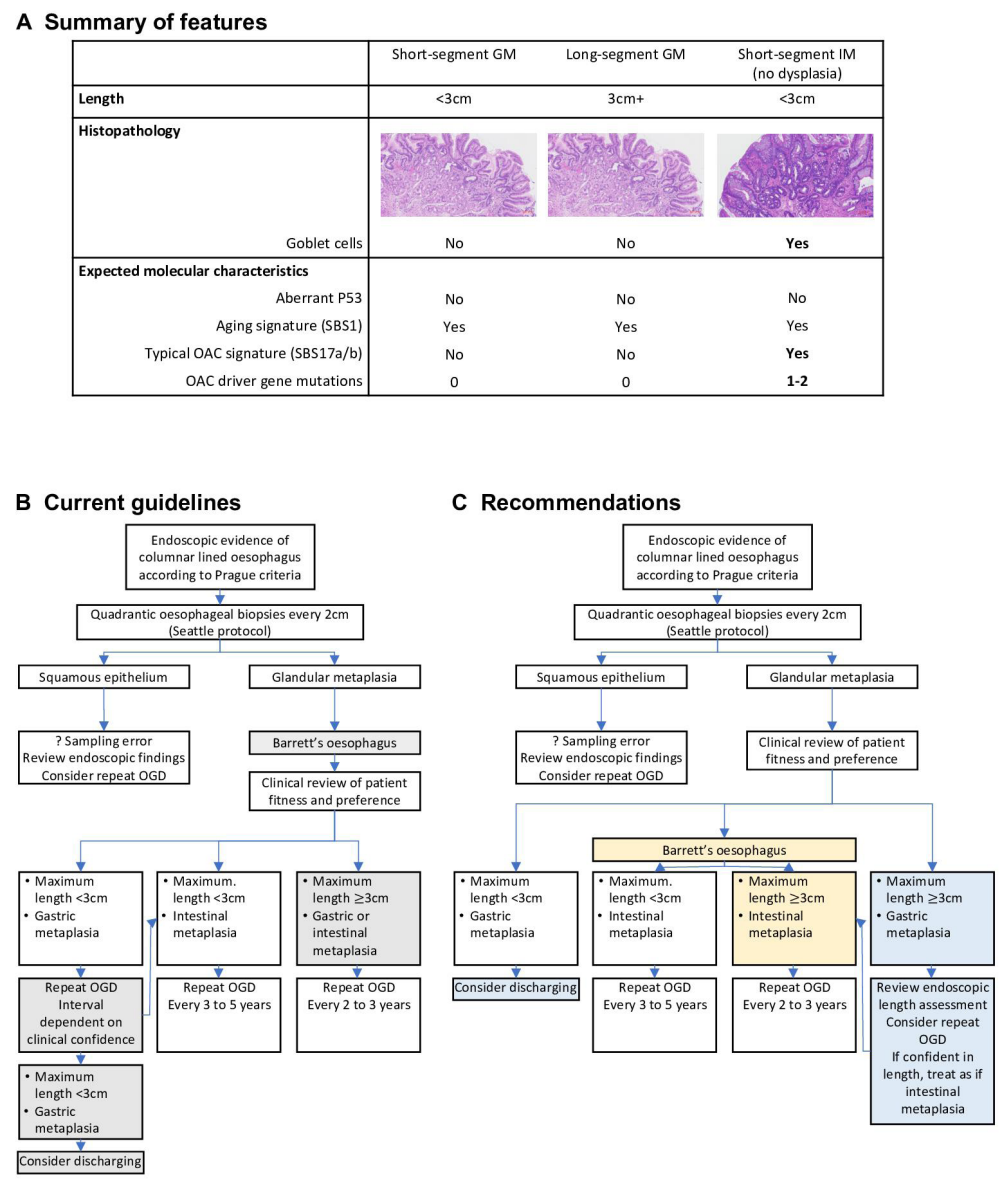
Short-segment gastric metaplasia does not warrant surveillance. (A) Summary of features of GM and IM. (B) Current British Society of Gastroenterology guidelines for management of non-dysplastic BO.^4^ Grey boxes denote areas of recommended change. (C) Recommended flow chart for non-dysplastic BO. Yellow boxes denote changes to BO definition, blue boxes denote changes to GM management. BO, Barrett’s oesophagus; GM, gastric metaplasia; IM, intestinal metaplasia; OAC, oesophageal adenocarcinoma; OGD, oesophagogastroduodenoscopy.

In summary, SS-GM is molecularly distinct from SS-IM. The malignant potential of SS-GM is low and lower than that of IM. We have suggested an alternative framework to help determine who should enter surveillance and would recommend that patients with SS-GM do not enter surveillance (figure 7). While some of these cases may have later been found to be GM+IM, short-segment GM+IM cases still represent low-risk cases. By contrast, there is high likelihood that LS-GM is undersampled GM+IM, and risk of progression is higher in longer cases.^42^ Therefore, in the absence of molecular confirmation of GM and reconfirmation of length, we would recommend these relatively rare cases be treated as if they were IM (figure 7).

## Data Availability

Data are available upon reasonable request. The sequencing data included in this study have been submitted to European Genome-phenome Archive (EGA; https://ega-archive.org/) with accession numbers EGAD00001011188, EGAD00001011187 and EGAD00001011255.
