# Supplementary material for: Understanding the malignant potential of gastric metaplasia of the oesophagus and its relevance to Barrett’s oesophagus surveillance: individual-level data analysis
Source: Gut. 2023 Nov 21;73(5):729–40. doi: 10.1136/gutjnl-2023-330721 (PMC11041591; doi:10.1136/gutjnl-2023-330721)
Supplement: Supplementary data [file gutjnl-2023-330721supp001.pdf]

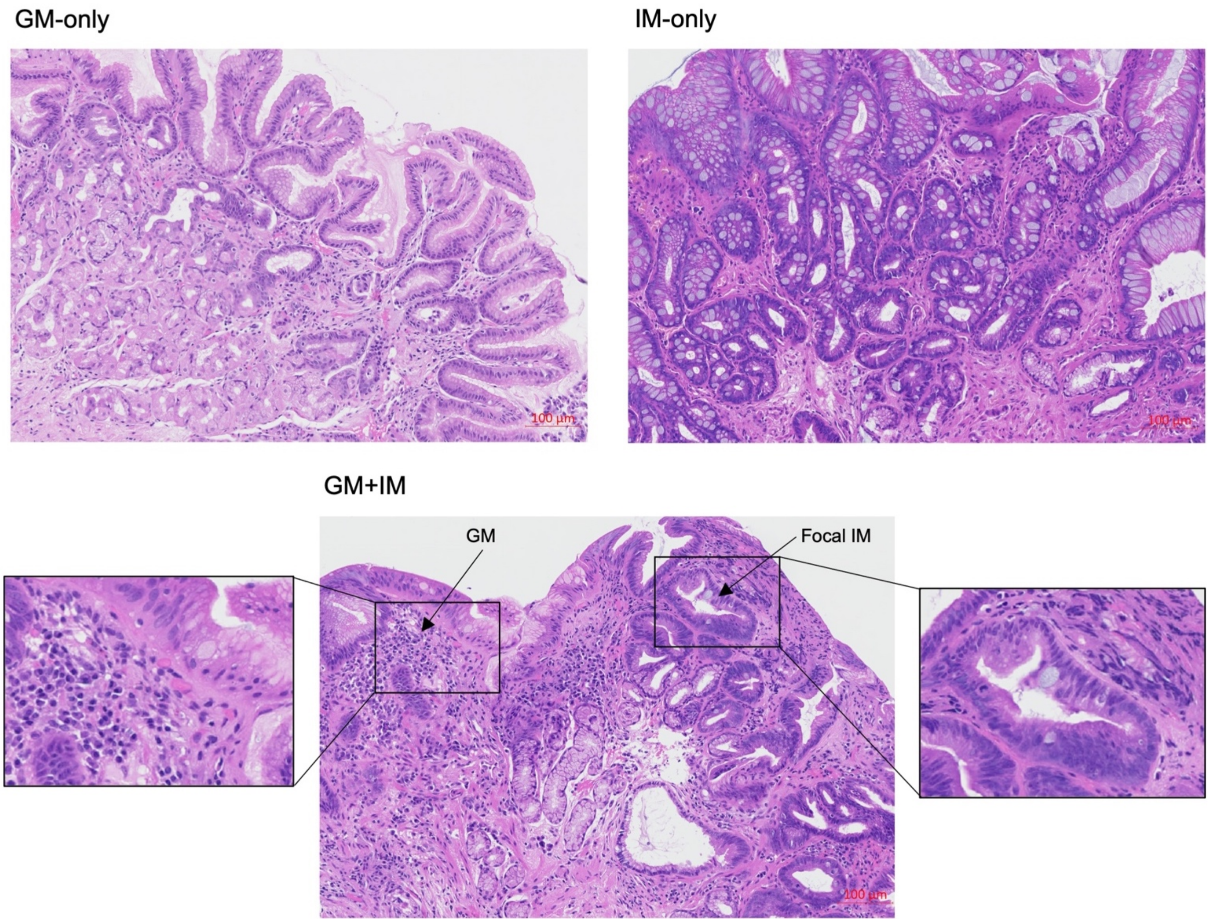

Supplementary Figure 1: Representative histopathology of GM-only, GM+IM and IM-only columnar lined oesophageal epithelium. IM is characterised by the presence of goblet cells. Example areas of GM and focal IM are indicated on the GM+IM image. All three main images are shown at the same level of magnification. GM, gastric metaplasia; IM, intestinal metaplasia.

Black EL, *et al. Gut* 2024; 73:729–740. doi: 10.1136/gutjnl-2023-330721

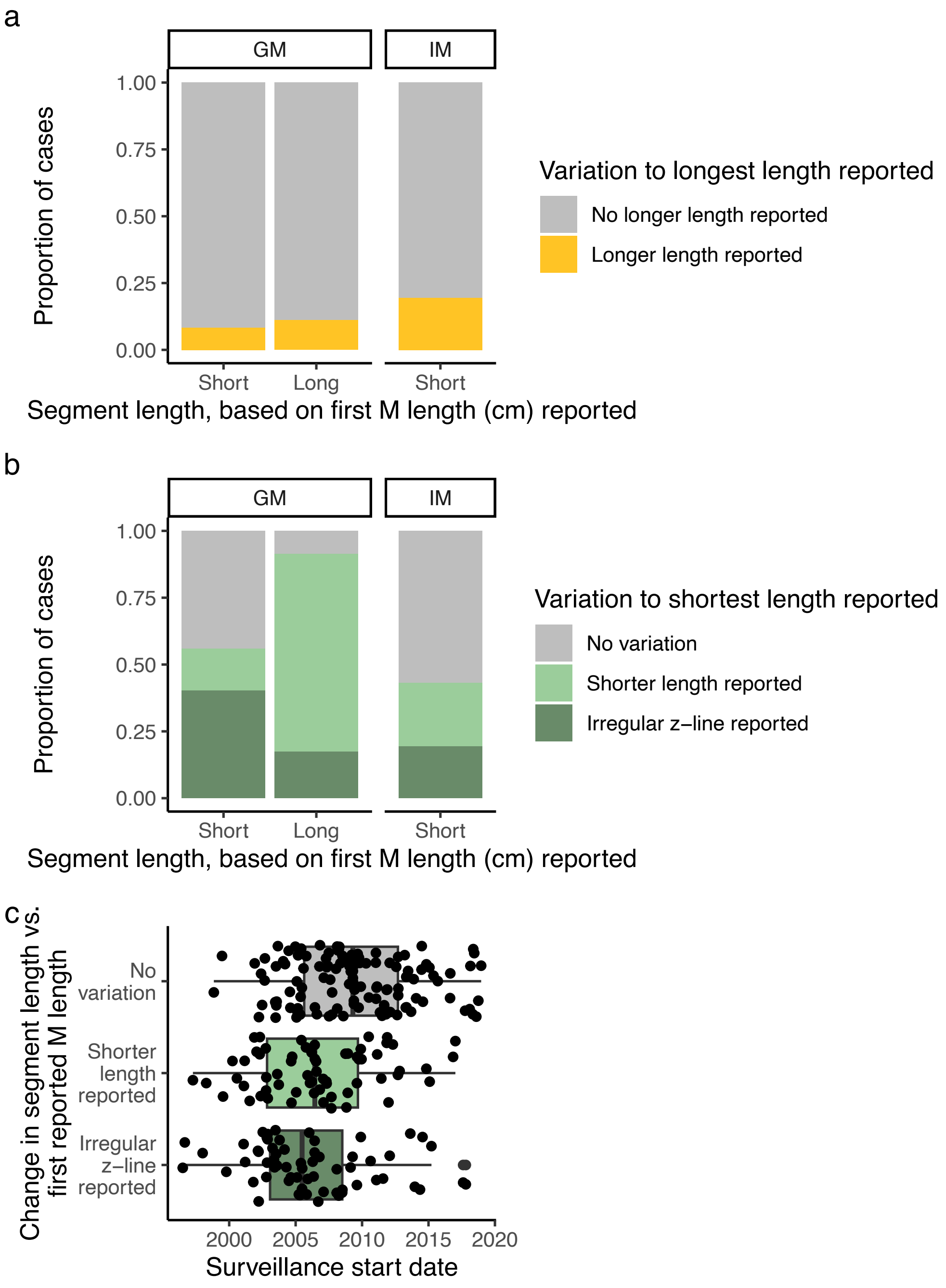

Supplementary Figure 2: Variation in length in subsequent OGDs. Proportion of cases with a) shorter or b) longer lengths than index-length reported in subsequent OGDs. It is possible for a case to have a shorter length reported in one follow-up and a longer length reported in a different follow-up, so the two are not mutually exclusive. c) Surveillance start date for segments with shorter lengths or irregular z-line reported, compared with those with no shorter length reported. GM, gastric metaplasia; IM, intestinal metaplasia; OGD, oesophagogastroduodenoscopy.

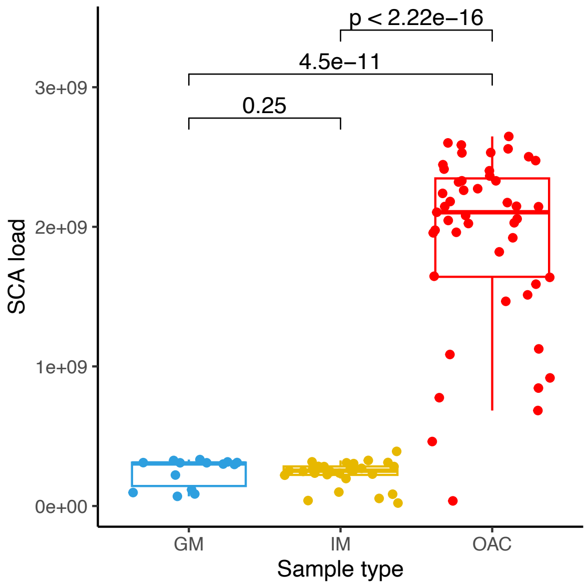

Supplementary Figure 3: SCA load comparison between OAC, GM and IM. SCA load taken as the length of genome altered by a copy gain, loss or copy neutral loss of heterozygosity; GM, gastric metaplasia; IM, intestinal metaplasia; OAC, oesophageal adenocarcinoma; SCA, somatic chromosomal alteration.

Black EL, *et al. Gut* 2024; 73:729–740. doi: 10.1136/gutjnl-2023-330721

a) Copy number events in driver genes, WGS samples

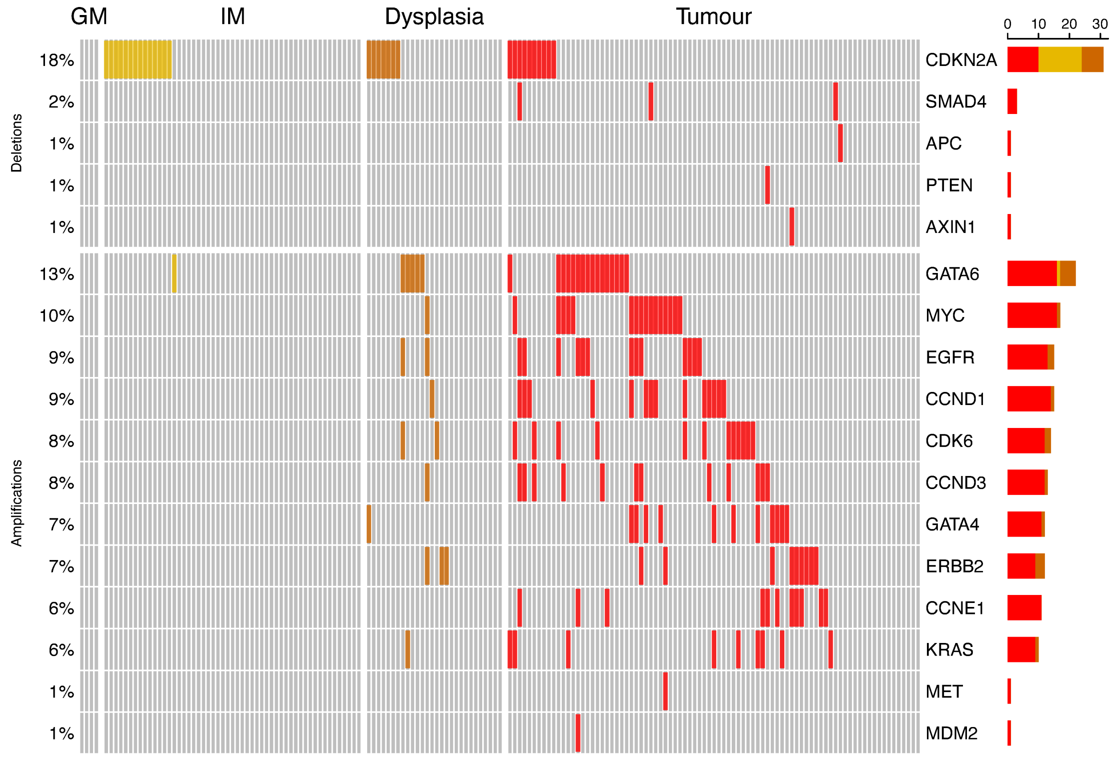

b) Copy number events in driver genes, WES samples

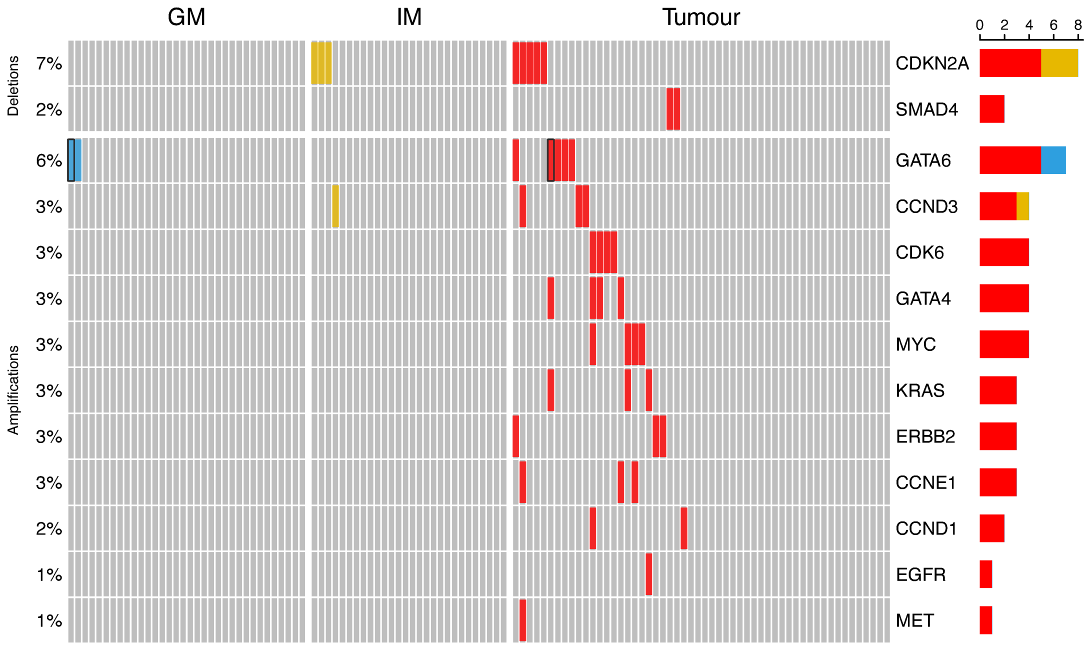

Supplementary Figure 4: Amplifications and deletions in copy number driver genes, in a) WGS samples and b) WES samples. Shared event between GM and Tumour is highlighted with a black outline. WGS, whole genome sequencing; WES, whole exome sequencing; GM, gastric metaplasia; IM, intestinal metaplasia.

a) Whole slide

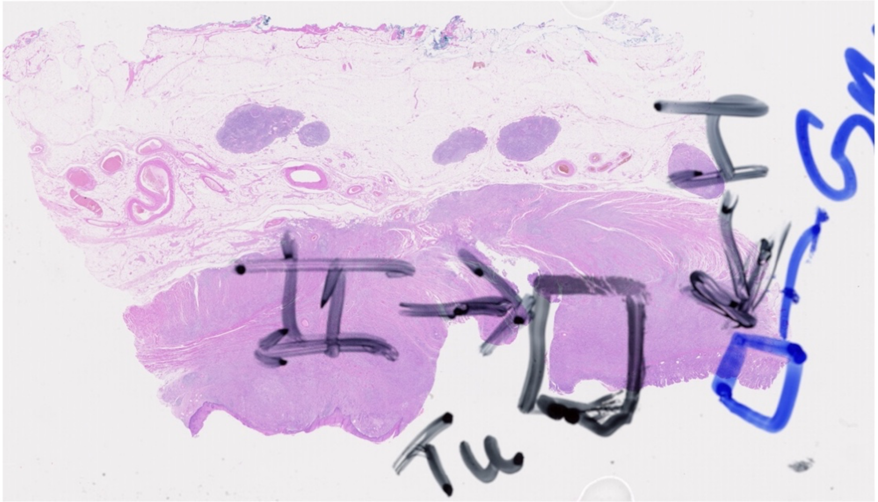

b) Part of Tumour (Black - Tu) region

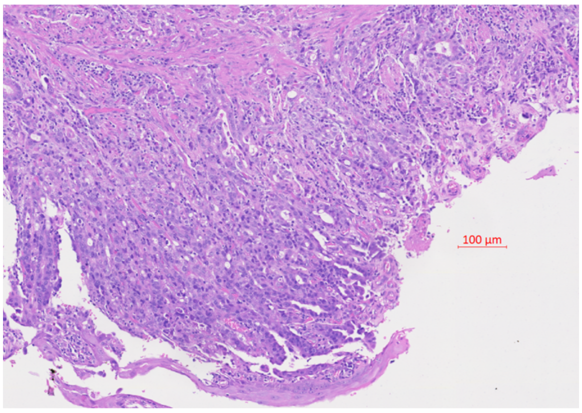

c) Part of gastric metaplasia (Blue – GM) region

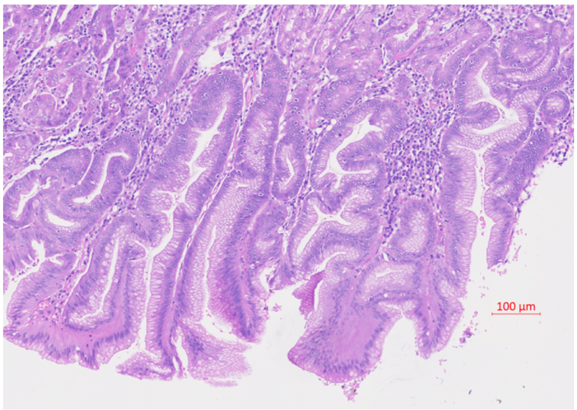

Supplementary Figure 5: Example of distinct tissue types identified by pathologists, which were then macro-dissected for sequencing. GM area is marked by blue box labelled 'GM' and 'I', tumour area is marked by black box labelled 'Tu' and 'II'. GM, gastric metaplasia.

Black EL, *et al. Gut* 2024; 73:729–740. doi: 10.1136/gutjnl-2023-330721

**Criterion type**	**Criterion**	**Starting number**	**# failing criterion**	**# after criterion applied**
Pathology	Must have at least two complete pathology records	5,625	3,744	1,881
Pathology	Pathology records must cover more than a year	1,881	401	1,480
Pathology	First pathology record must be GM or IM, or IND with subsequent GM	1,480	697	783
Pathology	First pathology record must clearly confirm and distinguish GM and IM	783	39	744
Pathology	No change in diagnosis within first year	744	3	741
Pathology	Not missing most recent pathology report	741	1	740
Endoscopy	Length data available from at least one report	740	20	720
Endoscopy	Length data available from a report matching index pathology	720	40	680
Endoscopy	Index length SS GM/IM or LS GM, including not z-line	680	421	259
Medical	No other cancer	259	9	250
Medical	No prior treatment for BO/OAC	250	5	245
Final checks	Length data conceivably consistent between endoscopies	245	1	244

Supplementary Table S1: Inclusion criteria for clinical outcomes cohort. GM, gastric metaplasia; IM, intestinal metaplasia; SS, short segment; LS, long segment; IND, indefinite for dysplasia; BO, Barrett's oesophagus; OAC, oesophageal adenocarcinoma.

Black EL, *et al. Gut* 2024; 73:729–740. doi: 10.1136/gutjnl-2023-330721

PatientID	BOType	Sex	Age	TStage	NStage	MStage	IM present (dysplasia)
P1	GM	F	59	T3	N0	M0	
P2	GM	M	78	T1b	N2	Mx	
P3	GM	F	69	T3	N2	M0	
P4	GM	M	66	T1a	N0	M0	
P5	GM	F	82	T1b	N0	M0	
P6	GM	F	81	T3	N2	M0	
P7	GM	M	78	T1a	N0	M0	
P8	GM	M	80	T2	N0	M0	
P9	GM	M	80	T1b	N0	M0	
P10	GM	M	71	T3	N1	M0	
P11	GM	F	73	T3	N0	M0	
P12	GM	M	72	T3	N3	M0	
P13	GM	M	65	T4a	N3	M0	
P14	GM	M	75	T3	N3	M0	
P15	GM	M	77	T3	N2	M0	
P16	GM	F	80	T3	N1	M0	
P17	GM	M	58	T2	N1	Mx	
P18	GM	M	77	T3	N1	Mx	
P19	GM	M	58	T2	N0	M0	
P20	GM	M	52	T1b	N0	M0	
P21	GM	M	78	T3	N1	M0	
P22	GM	M	63	T3	N2	M0	
P23	GM	M	67	T2	N0	M0	
P24	GM	M	74	T2	N0	M0	
P25	GM	M	72	T3	N3	M1	
P26	GM	M	76	T1a	N0	Mx	
P27	GM	M	56	T0	N0	M0	
P28	GM	M	57	T0	N0	M0	
P29	GM	M	68	T1a	N0	Mx	
P30	GM	F	86	T2	N1	M0	
P31	GM, IM	M	78	T3	N2	M0	
P32	GM, IM	M	74	T1a	N1	Mx	
P33	GM, IM	M	83	T2	N1	Mx	
P34	GM, IM	M	82	T2	N0	Mx	
P35	GM, IM	M	73	T2	N0	M0	
P36	GM, IM	M	62	T2	N0	M0	
P37	GM, IM	M	79	T3	N2	M0	
P38	GM, IM	M	70	T2	N1	Mx	
P39	HGD	M	50	T3	N3	Mx	Not recorded
P40	HGD	M	67	T1	N0	Mx	Not recorded
P41	HGD	M	76	T2	N1	M0	Yes
P42	HGD	M	69	T1b	N0	Mx	Yes
P43	HGD	F	66	T3	N1	Mx	Yes
P44	HGD	M	74	T3	N1	M0	Yes

Black EL, *et al. Gut* 2024; 73:729–740. doi: 10.1136/gutjnl-2023-330721

PatientID	BOType	Sex	Age	TStage	NStage	MStage	IM present (dysplasia)
P45	HGD	M	66	T3	N0	Mx	Yes
P46	HGD	M	60	T1a	N0	M0	Yes
P47	HGD	M	74	T1b	N0	Mx	Not recorded
P48	HGD	M	87	T3	N1	M0	Not recorded
P49	HGD	M	67	T3	N1	M0	Not recorded
P50	IM	M	71	T1a	N0	M0	
P51	IM	M	72	T1b	N0	M0	
P52	IM	F	52	T1b	N0	M0	
P52	LGD	F	52	T1b	N0	M0	Yes
P53	IM	M	80	T2	N0	M0	
P54	IM	M	82	T4a	N3	M0	
P55	IM	M	70	T1	N0	M0	
P56	IM	M	65	T3	N1	M0	
P57	IM	M	82	T3	N3	Mx	
P58	IM	M	83	T3	N2	Mx	
P59	IM	M	56	T1b	N0	Mx	
P60	IM	M	Unknown	T2	N0	M0	
P61	IM	M	85	T1b	N0	Mx	
P62	IM	M	54	T1a	N0	M0	
P63	IM	M	83	T2	N0	M0	
P64	IM	M	76	T2	N0	M0	
P65	IM	M	69	T1b	N1	M0	
P66	IM	M	74	T2	N0	Mx	
P67	IM	M	79	T1b	N0	M0	
P68	IM	F	80	T3	N0	M0	
P69	IM	M	77	T3	N2	Mx	
P70	IM	F	80	T1b	N2	M0	
P71	IM	F	79	T2	N1	M0	
P72	IM	M	74	T3	N0	Mx	
P73	IM	M	65	T1	N0	Mx	
P74	IM	M	69	T1b	N0	Mx	
P75	IM	M	68	T3	N3	M0	
P76	IM	M	84	T3	N1	M1	
P77	IM	F	50	T1b	N1	Mx	
P78	IM	F	62	T3	N0	M0	
P79	IM	M	58	T4a	N3	M1	
P80	IM	M	66	T3	N1	Mx	
P81	IM	M	76	T3	N3	M1	
P82	IM	M	73	T3	N3	M1	
P83	IM	M	62	T3	N3	M0	
P84	IM	M	67	T3	N2	M0	
P85	IM	M	61	T2	N1	M1	
P86	IM	M	79	T3	N1	M0	
P87	IM	M	66	T2	N0	Mx	

Black EL, *et al. Gut* 2024; 73:729–740. doi: 10.1136/gutjnl-2023-330721

PatientID	BOType	Sex	Age	TStage	NStage	MStage	IM present (dysplasia)
P88	IM	M	82	T3	N0	Mx	
P89	IM	M	59	T1b	N0	M0	
P90	IM	F	58	T1a	N0	M0	
P91	IM	M	68	T1b	N0	Mx	
P92	IM	F	56	T2	N1	Mx	
P93	IM	M	61	T2	N1	Mx	
P94	IM	M	83	T3	N2	Mx	
P95	IM	M	57	T3	N0	Mx	
P96	IM	M	71	T1a	N0	Mx	
P97	IM	M	61	T3	N2	Mx	
P98	IM	M	66	T3	N1	M0	
P99	IM	M	66	T1	N1	Mx	
P100	IM	M	58	T3	N2	M0	
P101	IM	M	67	T3	N2	M1	
P102	IM	M	63	T1	N0	Mx	
P103	IM	M	75	T3	N3	Mx	
P104	IM	M	75	T1a	N0	Mx	
P105	IM	M	72	T1b	N0	M0	
P106	IM	M	77	T2	N0	M0	
P107	IM	M	74	T3	N3	M0	
P108	IM	M	67	T3	N0	M0	
P109	IM	M	71	T3	N1	M0	
P110	IM	M	76	T2	N0	Mx	
P111	IM	M	58	T2	N1	M0	
P112	IM	M	71	T2	N1	M0	
P113	IM	M	85	T2	N0	M0	
P114	IM	M	69	T1b	N1	Mx	
P115	IM	M	82	T3	N0	M0	
P116	IM	M	59	T3	N1	Mx	
P117	IM	M	67	T2	N0	Mx	
P118	IM	M	61	T1b	N1	Mx	
P119	IM	M	67	Tx	N2	M1	
P120	IM	M	56	T3	N2	Mx	
P121	IM	M	83	T1b	N0	Mx	
P122	IM	M	54	T2	N1	M0	
P123	LGD	M	65	Tx	Nx	Mx	Yes
P124	LGD	M	62	T3	N2	M1	Not recorded
P125	LGD	M	79	T1b	N0	Mx	Yes
P126	LGD	M	83	T3	N2	Mx	Yes
P127	LGD	M	76	T1b	N0	M0	Yes
P128	LGD	F	58	T3	N0	M0	No
P129	LGD	M	73	T3	N1	M0	Yes
P130	LGD	M	64	T1a	N0	M0	Yes
P131	LGD	M	64	T0	N0	M0	No

**PatientID**	**BOType**	**Sex**	**Age**	**TStage**	**NStage**	**MStage**	**IM present (dysplasia)**
P132	LGD	F	84	T3	N1	M0	No
P133	LGD	M	73	T1b	N0	M0	Not recorded
P134	LGD	M	56	T1b	N0	M0	Not recorded
P135	LGD	M	80	T3	N3	M1	Not recorded
P136	LGD	M	79	T3	N0	M0	Not recorded
P137	LGD	M	63	T2	N0	Mx	Not recorded
P138	LGD	M	72	T3	N3	M0	Not recorded

Supplementary Table S2: Characteristics of patients with BO adjacent to OAC. Age is age at diagnosis. BO, Barrett's oesophagus; OAC, oesophageal adenocarcinoma; GM, gastric metaplasia; IM, intestinal metaplasia; LGD, low grade dysplasia; HGD, high grade dysplasia.

Black EL, *et al. Gut* 2024; 73:729–740. doi: 10.1136/gutjnl-2023-330721

All biopsy results	Total	Focal IM	Widespread IM	Unspecified IM
GM+IM (including progressors)	77	51	5	21
IM only (including progressors)	100	39	25	36
	Chi-squared test	p = 0.0003283		

Supplementary Table S3: Incidence of reporting of focal and widespread IM across the GM+IM and IM only cases. GM, gastric metaplasia; IM, intestinal metaplasia.

Index	Outcome	Total surveillance time		Time to first IM OGD	
		Mean (years)	Median (years)	Mean (years)	Median (years)
GM	GM-only	4.60	4.00	--	--
GM	GM+IM	9.75	10.26	3.86	3.09

Supplementary Table S4: Total surveillance time for all index-GM cases, and time to first IM OGD for index-GM, GM+IM outcome cases. GM, gastric metaplasia; IM, intestinal metaplasia; OGD, oesophagogastroduodenoscopy.

Black EL, *et al. Gut* 2024; 73:729–740. doi: 10.1136/gutjnl-2023-330721

SUPPLEMENTARY METHODS

DNA extraction for WES samples

FFPE slides were deparaffinised and samples then macro-dissected using a needle, to maximise cellularity. Areas measuring approximately 0.5x0.5cm were extracted. An extraction was made of each type of tissue present in sufficient size (OAC, IM or GM), with the IM and GM having to be adjacent to the OAC. Qiagen's Gene Read DNA FFPE kit was used. Blood germline controls were used for all but one case. For this one exception case, the germline control was snap-frozen, normal tissue taken distant from the tumour.

For most blood samples, DNA was extracted using the QIAamp Blood Maxi Kit (Qiagen). The following adaptations were made to the manufacturer's instructions: spin speeds were changed to 2400 rpm for steps 6, 7, 9-11, and 13; spinning times were changed to 6 min, 2 min, 30 min, 4 min, and 10 min, for steps 6,7, 9-11 and 13, respectively; samples were eluted in buffer AE. For a small number of blood samples, DNA was extracted from buffy coats using the QIAamp DNA Mini Kit (Qiagen), with no modifications to the manufacturer's instructions.

WES mutation filtering

Mutations called in the WES samples had to: fall within exonic regions or splicing regions of coding genes; not present as SNPs in 0.01% or more of the European and World populations database [Supp. Ref 1]; have at least 5 supporting reads; and variant allele frequency of at least 0.05. Additional filtering was performed to address artefact and quality issues associated with FFPE. Specifically**,** the average fragment length associated with a mutation had to be at least 85bp. If a mutation was present in at least 5 patients (5% of the cohort), the median distance of the mutation from the end of the read had to have a median average deviation of more than 1, and the active region the mutation occurred in had to have a median number of candidate variants of no more than 1.

Black EL, *et al. Gut* 2024; 73:729–740. doi: 10.1136/gutjnl-2023-330721

WES copy number calling and copy number analysis

Copy number calling was performed using Sequenza [Supp. Ref 2], first using the 'full' breaks method, and then re-run using a minimum consistent segmentation across the samples from the same case. Amplifications and deletions were analysed in subsets of genes recurrently amplified (*KRAS, MYC, ERBB2, MET, GATA4, CCND1, GATA6, CDK6, EGFR, CCNE1, CCND3, MDM2, PPM1D*) or deleted (*CDKN2A, PTEN, SMAD4, AXIN1, APC*) in OAC. To call an amplification, the total copy number had to be ≥5 for samples with no WGD and ≥9 for samples with WGD. WGD was called as per the PCAWG approach [Supp. Ref 3]. Deletions had total copy number of 0. To minimise false positives, amplifications or deletions had to also be called by VarScan2 [Supp. Ref 4] and amplifications had to have a segment length of at least 100kb.

SUPPLEMENTARY REFERENCES

[1] The 1000 Genomes Project Consortium. A global reference for human genetic variation. *Nature* 526:68–74, 2015. doi: 10.1038/nature15393.

[2] Favero F, Joshi T, Marquard AM et al. Sequenza: allele-specific copy number and mutation profiles from tumor sequencing data. *Annals of Oncol*ogy, 26(1):64-70, 2015. doi: 10.1093/annonc/mdu479.

[3] Dentro SC, Leshchiner I, Haase K et al. Characterizing genetic intra-tumor heterogeneity across 2,658 human cancer genomes. *Cell*, 184(8):2239-2254.e39, 2021. doi: 10.1016/j.cell.2021.03.009.

[4] Koboldt DC, Zhang Q, Larson DE et al. VarScan 2: somatic mutation and copy number alteration discovery in cancer by exome sequencing. *Genome research* 22(3):568-76, 2012. doi:10.1101/gr.129684.111
